# The Epithelial–Mesenchymal Transition Influences the Resistance of Oral Squamous Cell Carcinoma to Monoclonal Antibodies via Its Effect on Energy Homeostasis and the Tumor Microenvironment

**DOI:** 10.3390/cancers13235905

**Published:** 2021-11-24

**Authors:** Yunpeng Bai, Jingjing Sha, Tatsuo Okui, Ichiro Moriyama, Huy Xuan Ngo, Hiroto Tatsumi, Takahiro Kanno

**Affiliations:** 1 Department of Oral and Maxillofacial Surgery, Shimane University Faculty of Medicine, Izumo, Shimane 693-8501, Japan; xyywq@126.com (Y.B.); jsswjbnjw@gmail.com (J.S.); tokui@med.shimane-u.ac.jp (T.O.); ngoxuanhuy158@gmail.com (H.X.N.); tatsumi@med.shimane-u.ac.jp (H.T.); 2 Department of Medical Oncology/Innovative Cancer Center, Shimane University Hospital, Izumo, Shimane 693-8501, Japan; ichimori@med.shimane-u.ac.jp

**Keywords:** oral squamous cell carcinoma, chemotherapeutic monoclonal antibodies, cetuximab, pembrolizumab, nivolumab, epithelial–mesenchymal transition, tumor microenvironment

## Abstract

**Simple Summary:**

Developing therapeutic resistance to monoclonal antibodies (mAbs) causes increasing failure in oral squamous cell carcinoma (OSCC) treatment. A clear understanding of the molecular basis for drug resistance will pave the way for OSCC management and a new effective therapeutic modality. This review elucidates the role played by EMT during the emergence of mAbs resistance and the configuration of the tumor microenvironment. The cancer cells that undergo the EMT process also cause significant energy substrate consumption which leads to a limited number and function of effective T-cells, eventually leading to immune evasion. This review firstly reveals the implicit crosstalk between the EMT, energy metabolism, and therapeutic resistance of mAbs. A focus on the rebalanced energy homeostasis in cancer cells and T-cells may provide a new perspective on the treatment of OSCC.

**Abstract:**

Oral squamous cell carcinoma (OSCC) is a major type of cancer that accounts for over 90% of all oral cancer cases. Recently developed evidence-based therapeutic regimens for OSCC based on monoclonal antibodies (mAbs), such as cetuximab, pembrolizumab, and nivolumab, have attracted considerable attention worldwide due to their high specificity, low toxicity, and low rates of intolerance. However, the efficacy of those three mAbs remains poor because of the low rate of responders and acquired resistance within a short period of time. The epithelial–mesenchymal transition (EMT) process is fundamental for OSCC growth and metastasis and is also responsible for the poor response to mAbs. During EMT, cancer cells consume abundant energy substrates and create an immunosuppressive tumor microenvironment to support their growth and evade T cells. In this review, we provide an overview of the complex roles of major substrates and signaling pathways involved in the development of therapeutic resistance in OSCC. In addition, we summarize potential therapeutic strategies that may help overcome this resistance. This review aims to help oral oncologists and researchers aiming to manage OSCC and establish new treatment modalities.

## 1. Introduction

Oral squamous cell carcinoma (OSCC), the main pathological type of oral cancer (OC), accounts for at least 90% of cases of oral malignancy [1]. Although there have been many developments in cancer treatment, the overall 5-year survival rate for OSCC remains low, at ~50–60% [2]. The standard treatments for OSCC are surgery, radiotherapy, and chemotherapy. Unfortunately, adjuvant therapies such as chemotherapy do not achieve satisfactory results, largely due to low target selectivity, severe side effects, and drug resistance. Numerous studies have demonstrated that cancer cells tend to develop chemoresistance after frequent use of high-dose chemotherapy [3].

Several phenomena, including the epithelial–mesenchymal transition (EMT) and the accumulation of cancer stem cells (CSCs) and cancer-associated fibroblasts (CAFs), are implicated in chemoresistance in OSCC [4]. The EMT is a common feature of various types of cancer and is closely associated with CSCs and CAFs. During the EMT, a change occurs in the molecular pathways of cancer cells, and gene reprogramming induces their proliferation and invasiveness. Invasive cancer cells acquire mesenchymal features by gradually losing cell polarity, tight intercellular junctions, and the ability to express epithelial markers [5]. Numerous EMT-activating transcription factors (EMT-ATFs) participate in this process, including Snail, TWIST, and ZEB. The EMT-ATFs specifically bind to the promoter of E-cadherin through E-boxes and eventually suppress the expression of the adherent junction protein E-cadherin [6]. The balance between the epithelial marker E-cadherin and mesenchymal markers plays a pivotal role in the dynamic EMT process, which affects subsequent cancer cell migration, metastasis, immune evasion, and resistance to chemotherapeutic agents [7].

While primary tumor cells undergo EMT, those cells create an optimal tumor microenvironment (TME) by secreting various cytokines and proteases to facilitate their survival and ability to evade the immune system. Stromal cells, which are affected by the TME, are also activated to release several factors that trigger the EMT in primary tumor cells [8]. Methods to inhibit the EMT and convert the TME into a normal stromal cell microenvironment are needed.

Another problem for conventional chemotherapy is that cancer cells share similarities with normal host cells, so it is difficult for therapeutic agents to achieve high levels of selective cytotoxicity. However, in the last decade, monoclonal antibodies (mAbs) have shown great potential for use against many hematological and solid cancers in humans [9]. The mAbs target cancer cells by specifically binding to cell surface antigens that are necessary for cell proliferation or differentiation, or for immune meditation. Examples of such antigens include the epidermal growth factor receptor (EGFR), programmed cell death protein 1 (PD-1), cytotoxic T-lymphocyte-associated protein 4 (CTLA-4), and vascular endothelial growth factor and its receptor (VEGF and VEGFR, respectively) [10,11,12].

According to the National Comprehensive Cancer Network, only three mAbs, cetuximab, pembrolizumab, and nivolumab, have been used as evidence-based therapeutic regimens of mAbs for treating OSCC [13]. Although this new therapeutic approach offers many advantages, it also has several limitations, including adverse effects and the development of resistance, in addition to the high cost of producing the antibodies [14]. The development of resistance against mAbs, also seen in some OSCC patients, particularly when used alone without other chemotherapeutic agents, remains problematic [15]; nevertheless, the engineering of mAbs represents a major milestone in cancer therapeutics. To address this issue, we focused on the molecular pathways, proteins, and genes that participate in the key processes that contribute to the development of resistance to mAbs in OSCC patients, including the EMT process and energy metabolic homeostasis. Furthermore, we proposed a hypothesis regarding the latent mechanisms involved in the development of drug resistance to facilitate the identification of potential treatment targets. We concluded that, with concerted global efforts, the breadth and quality of treatment approaches using mAbs should improve significantly. A more specific, less toxic, and more cost-effective mAb will benefit OSCC patients worldwide.

## 2. Cetuximab: A mAb and EGFR Inhibitor

### 2.1. Rapid Response of Cancer Cells to Cetuximab Treatment

Cetuximab, an IgG1 mAb, is an inhibitor of EGFR. It is used for the treatment of locoregionally advanced, metastatic, and recurrent cancers, including OSCC [16]. Compared to EGF, cetuximab has a 5-fold higher affinity for EGFR, so can bind to EGFR prior to EGF and block the interaction of the ligand with its receptor [17].

EGFR, also known as ErbB1 or HER1, is a transmembrane tyrosine kinase receptor that binds to the EGF family of extracellular protein ligands. EGF stimulates OSCC cells to undergo the EMT. Up to 90% of OSCC cells overexpress EGFR. Upregulated EGFR is associated with poor prognosis, chemoresistance, and radioresistance [18]. Cetuximab seemed to be a reasonable therapeutic strategy for OSCC, however, very few patients responded to the treatment, and virtually all responders developed resistance within a short period [19].

Based on the work of Kagohara et al. [20], a cetuximab-treated cell line developed an early transcriptional response (within one day), and EGFR inhibition therapy induced a significant transcriptional variation within five days compared to untreated cells. EGFR inhibition therapy disrupts the homeostasis of cancer cells; therefore, the cells spontaneously progress to a state with activated heterogenetic hallmark pathways to overcome EGFR inhibition. This could explain the immediate upregulation of vimentin seen when cells were treated with cetuximab [20].

**Figure 1:**
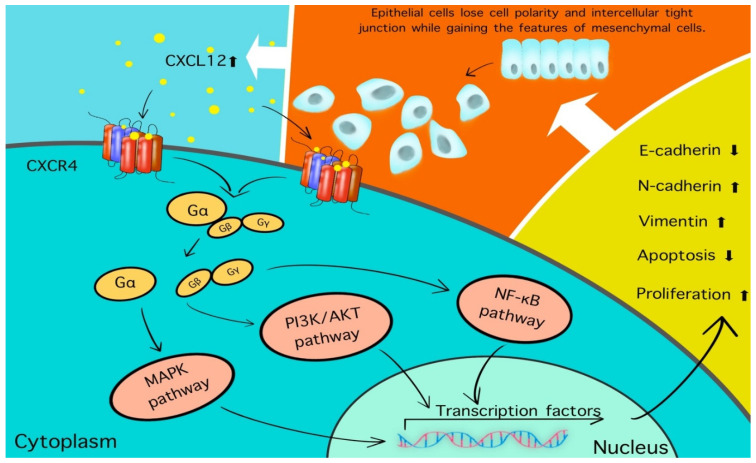
CXCR4, one of the receptors of CXCL12, is expressed on multiple cell types, including lymphocytes, hematopoietic stem cells, epithelial cells, and cancer cells [26]. The binding of CXCL12 to CXCR4 results in the activation of the CXCR4/CXCL12 axis and dissociation of the heterotrimeric protein complex (Gαβγ) to Gα and Gβγ subunits, which activate a number of signaling pathways including MAPK, PI3K/AKT, and NF-κB. Ultimately, cell growth, protein synthesis, and inflammation are upregulated. In addition to this change, the E-cadherin will be decreased and the expression of N-cadherin and vimentin will be raised; simultaneously, those cells lose cell polarity and tight intercellular junction while gaining the features of a mesenchymal cell and the capability of migration and invasion. This positive feedback loop stimulates more CXCL12 to be released into the TME to activate the CXCR4/CXCL12 axis.

As well as vimentin, other EMT-ATFs such as LEF1, TWIST1, and ZEB1 were significantly increased after treatment with cetuximab. Gene analysis of the same patients showed that Wnt, TGF-β, and NOTCH1 pathway-related genes were significantly upregulated following treatment with cetuximab. All of those genes were specifically involved in the extracellular matrix organization, including cell adhesion, migration, and the inflammatory response [21]. Five CAFs-associated genes, CXCL12, ACTA2, FAP, PDGFRB, and S100A4, were also significantly upregulated in the cetuximab-treated group compared to the control group. CXCL12, also known as stromal cell-derived factor-1α, binds to its receptor CXCR4 and activates the CXCR4/CXCL12 axis; this leads to tumor proliferation, vascularization, and metastasis [22]. Activation of the CXCR4/CXCL12 axis has been shown to decrease the levels of E-cadherin and increase those of vimentin and N-cadherin (Figure 1). These findings imply that cetuximab may induce the EMT indirectly, as well as CAFs proliferation, leading to the development of cancer cell resistance. The CXCR4 is expressed in multiple cell types, including lymphocytes, hematopoietic stem cells, epithelial cells, and cancer cells [23]. A recent study has demonstrated that CXCR4 may also play a role in CSCs in OSCC [24]. Inhibitors of the CXCR4/CXCL12 axis are currently under development [25].

### 2.2. Cetuximab Mainly Targets OSCC with Epithelial Features

In one study, cetuximab failed to block EGF-driven mesenchymal progression in transformed OSCC [27], suggesting a novel drug resistance mechanism. An earlier study reported that esophageal squamous cell carcinoma (ESCC) cells with mesenchymal features displayed resistance not only to cetuximab but also to tyrosine kinase inhibitors (TKIs) such as erlotinib, albeit that both drugs were highly effective against ESCC cells with epithelial features [28].

The progression of OSCC is always associated with the release of extracellular vesicles (EVs) from cancer cells into the milieu. The EVs usually contain CD9 and EGFR [29]. Earlier research showed that CD9-positive EVs were secreted more often by OSCC than epithelial cells [30]. Fujiwara et al. [31] reported that the transformation of normal epithelial cells into spindle phenotype cells triggered by EVs secreted by OSCC was blocked by cetuximab, both the expression of vimentin and levels of EGFR were also downregulated simultaneously. Therefore, we hypothesized that cetuximab primarily targets EV-secreting cancer cells, especially those with epithelial features rather than the completely transformed mesenchymal cancer cells. This phenomenon could lead to new therapeutic strategies.

Most anti-tumor drugs target a single oncogenic signal or phase. However, during the EMT, the signaling pathways and features of cancer cells are completely different among the “epithelial”, “mesenchymal”, and “transition” stages. Therefore, instead of using single drug treatment that inevitably results in the development of resistance or recurrence, multiple agents that tackle various pathways may yield better results.

### 2.3. Combination Treatment with Cetuximab

An effective regimen for refractory OSCC is the combination of cetuximab and cisplatin. Several studies have demonstrated that this combination is beneficial in terms of the tumor response and survival rate compared to single therapeutic agents [32,33].

It was proposed that cetuximab initiates cytoplasmic signaling through the autophosphorylation of intracellular domains [34]. Phosphorylated EGFR activated the MAPK and PI3K/AKT/mTOR pathways, which are also involved in the therapeutic effects of cisplatin [35]. However, cisplatin also induces cell apoptosis through the activation of the mitochondrial signaling pathway and AMP-activated protein kinase signaling pathway (AMPK) [36]. AMPK is a serine/threonine kinase that regulates intracellular energy homeostasis. The activation of the AMPK pathway could rapidly reprogram glucose metabolism, inducing cells to switch from active ATP consumption to ATP production to restore energy, potentially reversing the Warburg effect [37].

Cetuximab has been reported to have higher efficacy against K-RAS-type cancer cells than cells harboring a K-RAS mutation [38]. As a molecular switch, activated K-RAS is vital for stimulating the downstream signaling cascade via the Raf/MEK/ERK and PI3K/AKT/mTOR pathways after extracellular ligands, such as EGF, bind to the membrane receptor [39]. In OSCC patients, K-RAS frequently mutates into H-RAS, although the reason for this is unknown (this mutation is frequently detected in smokers and betel quid-chewing individuals [40]). Previous studies have demonstrated that the H-RAS mutation plays a crucial role in treatment failure and the development of resistance to cetuximab in OSCC, by stimulating downstream genes like c-MYC, BCL-XL, and BCL-2 [41]. Accordingly, we hypothesized that regulating AMPK levels may compensate for the cetuximab resistance caused by the K-RAS mutation. A study of colorectal cancer strongly supports this hypothesis [42], but whether the same mechanism occurs in OSCC remains to be proven.

The interaction between cetuximab and cisplatin may explain why these two drugs provide better outcomes in combination than either alone. Both drugs reciprocally affect the mitochondrial activity and energy homeostasis in cancer cells. During the EMT process, the cells require tremendous amounts of energy; to counteract the effect of the drugs, to switch from mitochondrial oxidative phosphorylation to cytosolic glycolysis to enable the EMT to be completed in a timely manner, and to build up sufficient biomass (by suppressing apoptosis and evading immunity) for resistance to anticancer therapy [43]. The Warburg effect may trigger the EMT and accelerate the glycolytic metabolism to generate sufficient ATP [44]. Lin et al. reported that when the respiratory enzyme citrate synthase (CS) was knocked down in HeLa cells, the expression of EMT-ATFs such as Snail and TWIST increased, and the MAPK signaling pathway was activated [45]. This EMT phenotype induced by CS knockdown was modulated by ATP treatment and partially reversed by the suppression of the MAPK pathway.

Altered energy homeostasis in association with the EMT may be responsible for resistance to cetuximab. Currently, there are no therapeutics specifically targeting the RAS mutation to restore energy homeostasis in cancer cells. An early study reported that tipifarnib, a farnesyltransferase inhibitor, competes with RAS and, thus, suppresses its activity; however, this effect is non-specific as other proteins such as centromeric proteins were also inhibited [46].

Although cetuximab has a strong anti-tumoral effect, only certain types of cancer cells respond to it. Unfortunately, OSCC patients with K-RAS mutations do not respond to cetuximab. However, combining cetuximab with other antitumor drugs greatly improves the overall response rate; therefore, oral oncologists should consider this strategy.

## 3. Immune Checkpoint Inhibitors

### 3.1. PD-1-Blocking Agents: Pembrolizumab and Nivolumab

One of the hallmarks of cancer is that the immune system does not mount an effective antitumor response [47]. The PD-1 receptor is mainly expressed on the surface of T cells [48]. When PD-1 binds to its ligands, including PD-L1 and PD-L2, the activation of cytotoxic T cells (CD8+ T cells) is inhibited [49]. Cancer cells can, thus, evade the T cell-induced antitumor activity. 

Pembrolizumab was developed to block the interaction of PD-1 with its ligands and inhibit immune evasion. Pembrolizumab is a highly selective monoclonal IgG4 isotype antibody that impedes inhibitory signals in T cells [50]. This antibody was first approved in the United States in 2014 [51] as a first-line treatment for metastatic bladder cancer patients with high levels of PD-L1 and resistance to cisplatin-based treatment. In patients with OSCC/head and neck squamous cell carcinoma (HNSCC), pembrolizumab is used as a second-line treatment after platinum-based chemotherapy. 

Nivolumab is another human IgG4 PD-1 immune checkpoint inhibitor antibody that blocks PD-L1 by binding to PD-1 [52]. Both pembrolizumab and nivolumab have been approved for patients with recurrent/metastatic OSCC who have previously undergone platinum-based chemotherapy [53]. 

As an immune checkpoint inhibitor, nivolumab can achieve similar clinical outcomes to pembrolizumab [54]. The two drugs have different affinity sites and bind to different epitopes of the same receptor; however, the difference in pharmacokinetics between these two drugs remains to be elucidated [55].

**Figure 2:**
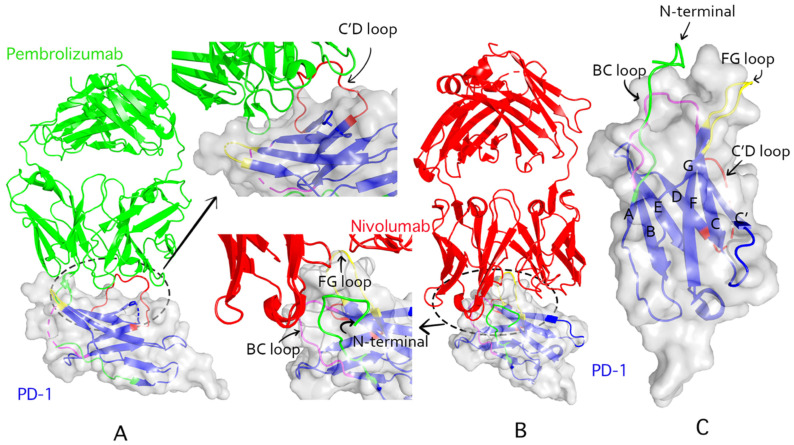
Structural interactions of pembrolizumab and nivolumab with PD-1. (**A**): Pembrolizumab complexed (PDB ID: 5jxe) with human PD-1 extracellular domain. The light green ribbons represent pembrolizumab. PD-1 is represented by the transparent blue ribbons. (**B**): The complex of nivolumab (PDB ID: 5wt9) and PD-1 extracellular domain; the nivolumab is represented by red ribbons. (**C**): The extracellular domain of human PD-1 (PDB ID: 3rrq). The BC loop, C’D loop, FG loop, and N-terminal domain are in magenta, red, yellow, and green colors, respectively.

PD-1 binding of pembrolizumab is dependent on a flexible C’D loop, while nivolumab targets PD-1 via the N-terminal extension and FG and BC loops (Figure 2). In a previous study of the crystal structure of pembrolizumab and nivolumab, the epitope region of the former was shown to have a much great affinity area to the PD-1 binding site than the latter [55]. Intriguingly, the binding sites of these two drugs on the PD-1 molecule do not overlap. These results imply that the two antibodies could act synergistically, thus, raising the question of whether they should be co-administered. Preclinical and early clinical data indicate that pembrolizumab and nivolumab can be used interchangeably [56].

### 3.2. EMT-ATFs Participate in the Configuration of an Immunosuppressive TME

Compared to conventional chemotherapy, the overall survival rate of all cancer patients receiving immunotherapy is significantly higher. However, 30–50% of patients remain unresponsive or less responsive to PD-1 blockade therapy [57].

A few patients initially responded to PD-1 blockade therapy but later became unresponsive, possibly due to insufficient effector T cells [58]. In another study, patients were unresponsive from the start of the treatment [59]. A recent study reported that in lung adenocarcinoma, the levels of multiple immune checkpoint molecules, including PD-L1, were increased, reflecting an EMT phenotype. This suggested that EMT could be associated with immunosuppressive TME changes [60,61]. The EMT-ATFs not only interact with the regulatory networks of microRNAs to promote cancer cell plasticity and maintain cancer stemness but also play an essential role in configuring the TME [62].

In the TME, Snail induces stromal cells to undergo the EMT, which increases the expression of immunosuppressive cells (such as CD4+ Foxp3+ Treg-like cells) [63]. Snail was also found to induce the upregulation of chemokines such as CCL2 and CXCL2, subsequently promoting the generation of immunosuppressive dendritic cells (DCs), Treg cell infiltration, and the inhibition of CD8+ T cells. Under hypoxic conditions, cancer cells can also secrete several chemokines that inhibit CD4+ and CD8+ T cell function [64], thus, establishing an immunosuppressive environment [65]. 

TWIST is another transcription factor with immunosuppressive activities. It upregulates PD-L1, which promotes the EMT and carcinogenesis in skin SCC [66]. TWIST was also reported to downregulate NF-κB and TNF-α, thereby regulating inflammatory suppression through type I interferons (IFNs) [67]. ZEB1 and ZEB2 were found to be involved in the activation of DCs and immune evasion in cancer [68].

### 3.3. Interactions among Energy Metabolism, Immunosuppression, and the TME

In addition to the EMT-ATFs, a complex regulatory network consisting of noncoding RNAs, epigenetic modifiers, post-translational regulators, and alternative splicing factors has been implicated in the dynamic regulation of the TME [69,70]. All of these extracellular factors promote a hypoxic, acidic, and inflammatory TME. Immune cells in the TME secrete cytokines, inflammatory factors, and chemokines to drive the EMT in cancer cells. In turn, cancer cells interact with immune cells to promote cell plasticity and the release of immunosuppressive substances or cytokines, which induce the infiltration of Treg cells or polarized M2 macrophages into the TME; therefore, an immunosuppressive microenvironment is generated, promoting cancer genesis and therapeutic resistance.

Cancer cells predominantly undergo glycolytic metabolism, which facilitates the rapid generation of ATP and inevitably leads to the accumulation of lactic acid in the milieu. T cells are similar to cancer cells in terms of their ability to recognize tumor antigens. To meet the bioenergetic demand for rapid proliferation, T cells also switch to glycolytic energy metabolism. Distinct from cancer cells, T cells produce pyruvic acid rather than lactic acid as the byproduct of glycolysis [71]. In accordance with the similar proliferation profile between cancer and T cells, the cells compete for energy sources and substrates for anabolic pathways. Several studies have demonstrated that glucose and tryptophan were excessively consumed by tumor cells, thereby restricting T cell glycolytic metabolism, activation, and proliferation, which eventually led to T cell dysfunction or depletion [72,73]. As a consequence, infiltration of T cells into the tumor mass was severely reduced. Furthermore, there was a decrease in the nuclear factor of activated T cells and natural killer (NK) cells, and in IFN-γ in the TME. These changes permitted cancer cells to escape from antitumor immunity [74].

Energy metabolism is mainly regulated by two completely opposing energy sensors: AMPK and mTOR [75]. The AMPK pathway regulates mitochondrial catabolism under restricted energy and nutrient conditions. However, the mTOR pathway (mainly mTOR complex 1, mTORC1) preferentially triggers anabolic metabolism [76,77]. Zhang et al. revealed that mTORC1 inhibitors activated the AMPK pathway, suppressed the EMT process, and downregulated PD-L1 expression in lung cancer [78]. In another report, the upregulated expression of PD-L1 was observed predominantly in patients with K-RAS mutations [79]. The overexpression of RAS promoted the progression of EMT by decreasing E-cadherin and increasing the expression of TGFβ, vimentin, and Snail, most likely via the MAPK and PI3K/AKT/mTOR pathways in concert with mTORC1.

Accordingly, we hypothesized that the upregulation of PD-L1 is correlated with cell energy metabolism (regulated by mTORC1) in the up and downstream pathways. Kumar et al. [58] suggested that enhanced activity in the mitochondrial metabolic pathway of CD8+ T cells may affect the PD-1 blockade. Therefore, it may be useful to explore ways to improve the precision of the mitochondrial function of CD8+ T-cells.

## 4. Summary

In 1997, rituximab became the first mAb to be approved for treating cancer and proven effective for the treatment of non-Hodgkin’s lymphoma. Following this success, numerous mAbs have been developed and used for the treatment of various human cancers [80]. Activated EGFR and its downstream signaling pathways were implicated in the proliferation, apoptosis suppression, invasion, and angiogenesis of cancer cells [81]. Compared to hematological cancers, solid tumors such as OSCC had more than 80% higher EGFR expression levels [82,83]. That may explain why cetuximab, as a novel treatment of OSCC, has garnered considerable attention since 2004 and has been approved for clinical use in Europe and the USA [84]. However, long-term use of cetuximab for OSCC often leads to drug resistance, and an improvement in the overall survival time of only 2.7 months compared to conventional chemotherapy alone [17,85,86].

In 2016, the FDA approved the use of pembrolizumab and nivolumab in patients with platinum-resistant OSCC; however, less than 20% of the patients responded well to those immunotherapies [87]. Moreover, approximately 50% of patients with OSCC are in a late stage of disease and the 5-year survival rate remains below 60% [88,89]. The treatment options for OSCC patients are limited, particularly for advanced patients who are refractory to chemotherapy. It is imperative to better understand the molecular mechanisms underlying drug resistance so that more effective therapies can be developed.

In OSCC, precancerous mutations may develop in cells remote from the original tumor [90]. This is known as field cancerization, whereby oral epithelial dysplasia and oral intraepithelial neoplasia patients may progress to OSCC [91]. Premalignant cells, over surgical margins, may develop into secondary tumors differing in clonality from the primary tumor [90].

Premalignant cells can trigger the EMT, and release chemokines and transcriptional factors to create a low-energy, hypoxic, and immunosuppressive TME to promote their survival. This environment then activates the AMPK pathway [92]. The activation of AMPK increases cell catabolism, and decreases anabolism, through the phosphorylation of key proteins in several pathways, including mTORC1 [93]. AMPK is an attractive therapeutic target in many diseases, including cancer and type II diabetes. Under energy-depleted conditions, the activation of AMPK leads to a decrease in mTORC1 activity, which retards cell growth and protein synthesis.

**Figure 3:**
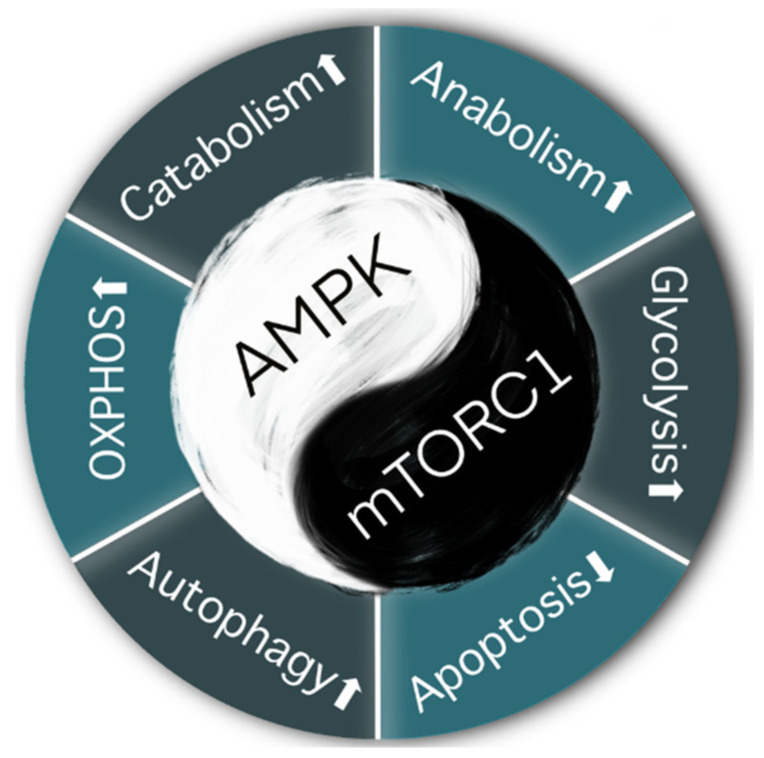
AMPK and mTORC1 are components of conserved pathways that evolved via a yin-yang-like antagonistic mechanism controlling catabolism and anabolism [94]. The AMPK pathway prompts cell autophagy and regulates catabolic metabolism and oxidative phosphorylation (OXPHOS) under restricted nutrient and energy conditions. The mTOCR1 pathway preferentially activates anabolic metabolism and glycolysis, while suppressing cell apoptosis [58].

**Figure 4:**
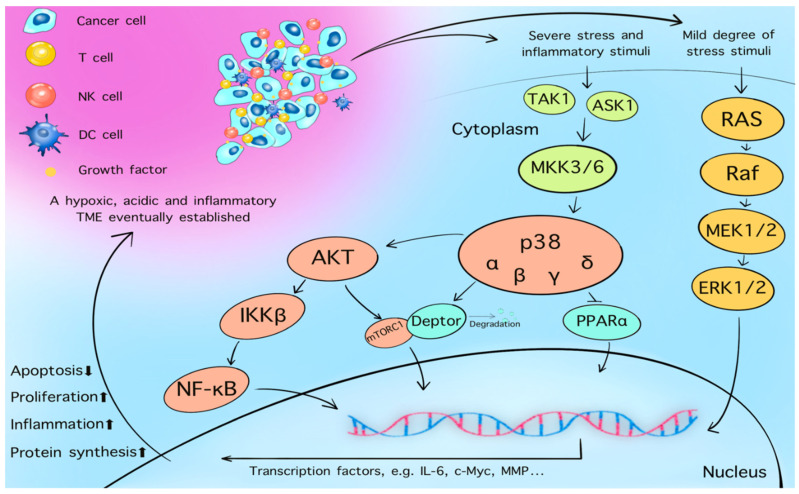
Four p38 subfamilies have been identified: p38-α, -β, -γ, and -δ [96]. MAPK cascade is the main mechanism by which the four p38 isoforms are activated in human cells [97]. Protein kinase activity of p38 is stimulated by oncogenic TAK1 or ASK1 through its downstream mitogen-activated protein kinase kinases, MKK3 and MKK6, under conditions of severe stress and inflammation (under mild stress, the ERK/MAPK pathway is activated) [98]. Activated p38 also interacts with AKT and NF-κB, triggering downstream mTORC1 via the degradation of DEPTOR, or by suppressing the activation of PPARα [95]. These changes promote cell proliferation, inflammation, protein synthesis, and decrease apoptosis, ultimately leading to a hypoxic, acidic, and inflammatory TME.

The mTORC1 exerts opposing effects on AMPK (Figure 3) [94]. When growth factors such as EGF and VEGF bind to their tyrosine kinase receptor on the cell membrane, the PI3K/AKT pathway is activated. Furthermore, phosphoinositide-dependent kinase-1 activates AKT and mTORC1, which prompts the entire pathway to enhance cell proliferation. The MAPK pathway is also activated by these growth factors. Cytosolic glycolysis and protein synthesis are enhanced, which provides more energy and, thus, promotes cancer cell survival. As a member of the stress-activated protein kinase family, p38 is activated during the energy depletion phase [93]. Interactions among p38, AKT, and NF-κB, trigger the downstream expression of mTORC1, leading to an accumulation of reactive oxygen species (ROS) and DNA damage (Figure 4) [95].

The overall response rate to the EGFR inhibitor cetuximab varies dramatically among patients and cannot be predicted based on EGFR binding alone. Whether the downstream pathway is normally regulated by K-RAS, but not by another mutated RAS, is an important consideration. In OSCC patients, mutations are most common in K-RAS genes, including H-Ras and N-Ras; these mutations were detected in over 30% of patients with OSCC in Southeast Asia [99]. Mutated K-RAS genes can activate the MAPK and PI3K/AKT/mTOR pathways, and regulate the expression of mTORC1; ultimately, OSCC cells become resistant to cetuximab.

Such resistance can also develop to PD-1 blockers. Although pembrolizumab and nivolumab can both interact with PD-1 to activate T cells, higher energy consumption by CD8+ T cells and a lower concentration of energy substrates in the TME increase the anabolism of T cells; this mainly depends on the activation of the mTORC1 pathway. Increased p38 and ROS accumulation further damages the mitochondria in T cells, leading to an exhausted state [58]. The PD-1 blockade ultimately shortens the life span of T cells. Furthermore, the elimination of T cells from the cancer field (by over-activating them) may explain why most OSCC patients show an excellent initial response to pembrolizumab/nivolumab, but subsequently become unresponsive [100].

## 5. A potential Novel Combination Therapy

In this review, we showed that the EMT, which leads to a newly configured TME and altered cell homeostasis, must be considered when developing novel treatments for OSCC. Combining several anti-tumor drugs, which represents the current strategy, is not effective over the long term.

### 5.1. Combined Use of the Small-Molecule Inhibitors MEK

Blocking the PI3K/AKT/mTOR and MAPK pathways by small-molecule inhibitors may be effective for treating OSCC. To date, four small-molecule MEK inhibitors have been approved by the FDA: trametinib, selumetinib, cobimetinib, and binimetinib [101]. Several other inhibitors are also under development, such as PD184352 [102] and PD0325901 [103]. A recent open-label, three-arm phase III trial evaluated the safety and efficacy of encorafenib and cetuximab, with or without binimetinib, for treating patients with BRAF^V600E^ (a gene that encodes B-Raf)-mutated colorectal cancer. The overall response rates were 26%, 20%, and 2% for the triplet, doublet, and control arms, respectively. The progression-free survival times were 4.3, 4.2, and 1.5 months, respectively [104,105].

The combination of a MEK inhibitor and immunotherapy was associated with a satisfactory toxicity profile in BRAF^V600E^-mutant melanoma patients [106,107]. In a phase II trial of the triple combination of trametinib, dabrafenib, and pembrolizumab, the objective response rate was 63.3% (38 out of 60 patients with BRAF^V600E^-mutated advanced melanoma); 17 of these 38 patients (44.7%) had ongoing responses [108].

### 5.2. EGFR and PD-1 Inhibitors

#### 5.2.1. EGFR-TKIs with a PD-1 Inhibitor

Preclinical studies indicated that the activation of EGFR not only stimulated tumor growth but was positively correlated with the overexpression of PD-L1. Treatment of EGFR-mutant non-small cell lung cancer (NSCLC) cell lines with EGFR-TKIs decreased the expression of PD-1 and PD-L1 by inhibiting the NF-κB pathway [109,110,111]. These studies provided a theoretical basis for the combination treatment of EGFR-TKIs and immunotherapy. Haratani et al. reported that T790-negative patients with EGFR mutation-positive NSCLC were more likely to benefit from nivolumab after EGFR-TKI treatment [112]. This result was attributed to the fact that NSCLC patients with certain types of EGFR mutations may have a higher nonsynonymous mutation burden, such that they are more responsive to PD-1 inhibitors. However, it should be noted that the trial mainly included lung cancer patients rather than OSCC patients. OSCC is characterized by a higher tumor mutation burden, higher expression of PD-L1, and increased CD8+ T cell infiltration; therefore, OSCC patients can be expected to benefit more from combination treatment.

#### 5.2.2. EGFR-mAb with a PD-1 Inhibitor

A multi-center phase II study investigating the combination of the EGFR-mAb cetuximab and PD-1 inhibitor pembrolizumab was conducted from 2017 to 2019. The study focused on recurrent and metastatic HNSCC; in 45% of patients, the primary tumor site was in the oral cavity [113]. An overall response rate of 45% was achieved with combination therapy, which exceeded the response rates for pembrolizumab (16–18%) and cetuximab (6–13%) monotherapy [114]. We suggest that the resistance of cancer cells to cetuximab may be associated with a K-RAS mutation. The activation of the MAPK pathway enhanced the expression of PD-L1 and the infiltration of T cells and, thus, the response to immunotherapy. Although its mechanism of action is yet to be fully elucidated, this combination therapy has a promising future.

## 6. Conclusions and Perspectives

The use of two or more drugs for OSCC treatment inevitably leads to more severe side effects and immunotherapy can cause immune-related adverse events [115]. Therefore, combination therapy should be considered as an interim strategy while we wait for better treatments to be developed.

Recent studies aiming to characterize the interactions among the EMT, energy metabolism in cancer cells, immunity, and the TME have provided important results that should inform future research. By further exploring the components and sub-pathways of the MAPK, PI3K/AKT/mTOR, and AMPK pathways, as well as their interactions with other pathways, we should be able to further improve evidence-based therapies of mAbs for treating OSCC.
